# TRADD Mediates RIPK1-Independent Necroptosis Induced by Tumor Necrosis Factor

**DOI:** 10.3389/fcell.2019.00393

**Published:** 2020-01-22

**Authors:** Lili Wang, Xixi Chang, Jinli Feng, Jiyun Yu, Guozhu Chen

**Affiliations:** ^1^Institute of Military Cognition and Brain Sciences, Academy of Military Medical Sciences, Beijing, China; ^2^Department of Neurology, The Eighth Medical Center of Chinese PLA General Hospital, Beijing, China; ^3^Beijing Zhendandingtai Biotechnology Co., Ltd, Beijing, China

**Keywords:** TNF receptor type 1-associated death domain protein, receptor-interacting serine/threonine-protein kinase 1, receptor-interacting serine/threonine-protein kinase 3, necroptosis, tumor necrosis factor

## Abstract

As a programmed necrotic cell death, necroptosis has the intrinsic initiators, including receptor-interacting serine/threonine-protein kinase 1 (RIPK1), RIPK3 and mixed-lineage kinase domain-like protein (MLKL), which combine to form necroptotic signaling pathway and mediate necroptosis induced by various necroptotic stimuli, such as tumor necrosis factor (TNF). Although chemical inhibition of RIPK1 blocks TNF-induced necroptosis, genetic elimination of RIPK1 does not suppress but facilitate necroptosis triggered by TNF. Moreover, RIPK3 has been reported to mediate the RIPK1-independent necroptosis, but the involved mechanism is unclear. In this study, we found that TRADD was essential for TNF-induced necroptosis in RIPK1-knockdown L929 and HT-22 cells. Mechanistic study demonstrated that TRADD bound RIPK3 to form new protein complex, which then promoted RIPK3 phosphorylation via facilitating RIPK3 oligomerization, leading to RIPK3-MLKL signaling pathway activation. Therefore, TRADD acted as a partner of RIPK3 to initiate necroptosis in RIPK1-knockdown L929 and HT-22 cells in response to TNF stimulation. In addition, TRADD was critical for the accumulation of reactive oxygen species (ROS), which contributed to RIPK1-independent necroptosis triggered by TNF. Collectively, our data demonstrate that TRADD acts as the new target protein for TNF-induced RIPK3 activation and the subsequent necroptosis in a RIPK1-independent manner.

## Introduction

It is well known that programmed cell death has been classified into several distinct forms, including apoptosis, necroptosis, pyroptosis and ferroptosis (Ashkenazi and Salvesen, 2014; Su et al., 2015; Robinson et al., 2019). As a relatively newly discovered programmed cell death, necroptosis is essential for many kinds of physiological and pathological processes, including embryonic development (Zhang et al., 2011; Newton et al., 2016), tissue injury (Degterev et al., 2005; Zhang T. et al., 2016), inflammation (Pasparakis and Vandenabeele, 2015; Lin et al., 2016) and host defense (Mocarski et al., 2012; Nogusa et al., 2016). Many kinds of necroptotic stimuli have been identified to initiate necroptosis, such as death receptor ligands (Micheau and Tschopp, 2003; Kim et al., 2007), Toll-like receptor (TLR) 3 and TLR4 ligands (He et al., 2011; Takemura et al., 2015), virus infection sensors (Upton et al., 2012; Thapa et al., 2016; Upton and Kaiser, 2017) and interferons (Robinson et al., 2012; Thapa et al., 2013). Among the family of death receptor ligands, tumor necrosis factor (TNF) could bind its receptor (TNFR) to initiate necroptotic or apoptotic signaling transduction (Hitomi et al., 2008; Galluzzi and Kroemer, 2011). As TNF induces apoptosis through initiating the activation of caspase 8 and the downstream caspase signal pathway, Z-VAD-FMK (Z-VAD), the pan-caspase inhibitor, blocks TNF-induced apoptosis by suppressing caspase activation (Van Herreweghe et al., 2010). However, in some necroptotic cellular model, such as L929 cells, Z-VAD has been reported to promote TNF-induced necroptosis through inhibiting the proteolitic activity of caspase 8 (Vercammen et al., 1997, 1998). Therefore, necroptosis was usually triggered by TNF plus Z-VAD. In addition, TNF-induced L929 cell necroptosis has been extensively studied, and this cell line has been identified as a well-established necroptotic cellular model (Hitomi et al., 2008; Galluzzi and Kroemer, 2011).

As the firstly identified target protein in initiating necroptosis, receptor-interacting serine/threonine-protein kinase 1 (RIPK1) binds TNF receptor (TNFR) to form complex I via death domain upon TNFR ligation (Hsu et al., 1995, 1996). In addition, several other adaptor proteins contain death domain, including TRADD, FADD and TRAF2, were also recruited into complex I, which acts as the platform to activate the nuclear factor (NF)κB and MAPK signaling pathways (Green and Llambi, 2015; Peltzer et al., 2016). However, in cells destined to necroptosis, RIPK1 binds its partner receptor-interacting serine/threonine-protein kinase 3 (RIPK3) to form necrosome, which promotes activation of RIPK1 and RIPK3 via facilitating their phosphorylation (Cho et al., 2009; Li et al., 2012). RIPK3 then recruits its substrate protein mixed-lineage kinase domain-like protein (MLKL) to form protein complex, and promotes MLKL phosphorylation, oligomerization and membrane translocation, resulting in membrane disruption and the subsequent necroptosis (Galluzzi et al., 2014; Wang et al., 2014). In addition, phosphorylated RIPK3 also induce accumulation of cellular reactive oxygen species (ROS), which oxidize the disulfide bonds in the cysteine, leading to RIPK1 and MLKL oligomerization (Chen et al., 2014; Schenk and Fulda, 2015; Zhang et al., 2017). Therefore, RIPK1, RIPK3 and MLKL combine to form the new signaling pathway which is critical for initiating necroptosis in response to TNF stimulation (Newton, 2015; Zhang J. et al., 2016).

It has been reported that RIPK1 transfers necroptotic signals through binding TNFR1 or RIPK3 to form different protein complexes, such as protein Complex I and necrosome (Green et al., 2011; Silke et al., 2015). Moreover, the formation of necrosome enables RIPK3 in the necrosome to recruit free RIPK3 to form RIPK3 dimer or oligomer, which promotes RIPK3 autophosphorylation through intramolecular reaction (Orozco et al., 2014; Wu et al., 2014). Therefore, RIPK1 initiates RIPK3-MLKL signaling pathway activation in necroptotic signaling transduction. Although chemical inhibition of RIPK1 almost fully inhibits necroptosis induced by TNF, genetic elimination of RIPK1 does not inhibit, but promote necroptosis (Degterev et al., 2005; Moujalled et al., 2013; Kearney et al., 2014). Moreover, RIPK3 has been reported to mediate RIPK1-independent necroptosis (Kearney et al., 2014), but it remains unclear how to transduce necroptotic signals from TNFR1 to RIPK3 in the absence of RIPK1.

In the current study, TRADD was identified as the target protein for TNF-induced necroptosis in RIPK1-knockdown L929 and HT-22 cells. Mechanistic study discovered that TRADD bound RIPK3 to form new protein complex and activated RIPK3 via facilitating RIPK3 oligomerization and the subsequent autophosphorylation, leading to RIPK3-MLKL signaling pathway activation. Moreover, TRADD was also essential for ROS accumulation which also contributed to RIPK1-independent necroptosis in response to TNF stimulation. Therefore, our data demonstrate that TRADD acts as the partner of RIPK3 to initiate RIPK1-independent necroptosis upon TNFR ligation.

## Results

### RIPK3 Mediates Necroptosis Induced by TNF in RIPK1-Knockdown L929 Cells

**FIGURE 1:**
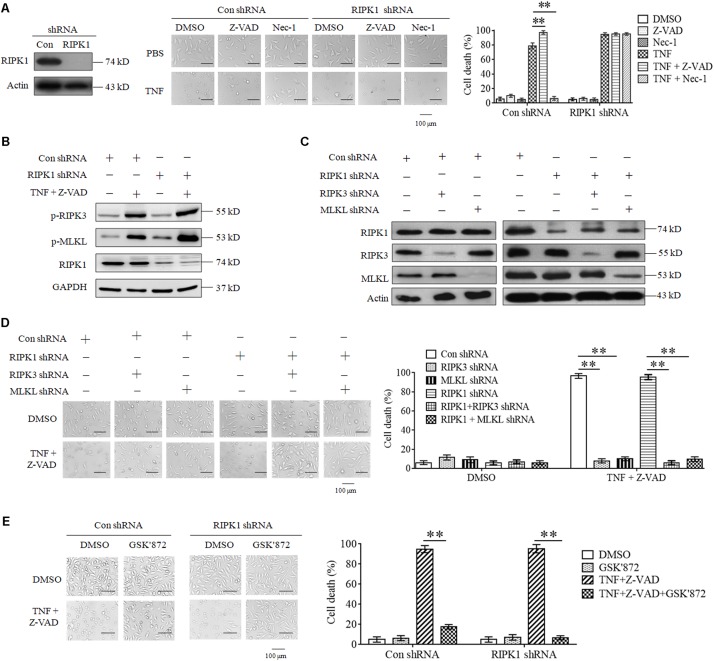
RIPK3 mediates TNF-induced necroptosis in RIPK1-knockdown L929 and HT-22 cells. **(A)**. TNF induces L929 cells necroptosis in RIPK1-dependent and independent manners. L929 cells were infected with RIPK1 shRNA or con shRNA lentivirus for 48 h and then treated with TNF in the absence or presence of Z-VAD or Nec-1 for another 48 h. Western blotting was performed to detect the knockdown efficiency of RIPK1. Actin was used as a loading control. Cell death was determined through microscopy (200× magnification) based on cellular morphologic changes. The proportion of propidium iodide-positive cells was measured via flow cytometry and used as the cell death value (%). ^∗∗^*P* < 0.01. **(B)** RIPK1 knockdown has no inhibitory effect on TNF-induced RIPK3 and MLKL phosphorylation. RIPK1 knockdown and the negative control L929 cells were treated with or without TNF plus Z-VAD for 3 h, and western blotting was used to detect the phosphorylation level of RIPK3 and MLKL (p-RIPK3 and p-MLKL) and the protein level of RIPK1. GAPDH was used as a loading control. **(C,D)** RIPK3 and MLKL mediate RIPK1-dependent and independent necroptosis triggered by TNF plus Z-VAD. L929 cells were infected with the indicated lentivirus, and western blotting was used to evaluate the knockdown efficiency. Actin was used as a loading control **(C)**. Cells were treated with or without TNF plus Z-VAD for 24 h, and the cell death was determined through microscopy (200× magnification) based on cellular morphologic changes and quantified by measuring the proportion of propidium iodide-positive cells **(D)**. ^∗∗^*P* < 0.01. **(E)** RIPK3 inhibitor protects L929 cells from necroptosis induced by TNF. RIPK1-knockdown and the negative control L929 cells were treated with TNF plus Z-VAD for 48 h in the absence or presence of GSK’872. Cell death was determined through microscopy (200 × magnification) based on cellular morphologic changes and quantified by measuring the proportion of propidium iodide-positive cells. ^∗∗^*P* < 0.01. In this and the following experiments, all the cell death determined by using microscopy was repeated three times independently, and three fields in each group were observed, and representative images are shown. In this and the following experiments, all the cell death values (%) are shown as the mean ±SD of three separate experiments (*n* = 3) and they were analyzed by a two-tailed *t*-test or ANOVA. All the western blot analyses in this and the following experiments were repeated independently three times, with representative images shown.

As the first target protein identified for necroptosis induction, RIPK1 binds its partner RIPK3 to form necrosome and initiates necroptosis in response to TNF stimulation (Cho et al., 2009; He et al., 2009). However, we found that RIPK1 knockdown did not inhibit L929 cell death induced by TNF (Figure 1A). Moreover, Z-VAD-FMK, the pan-caspase inhibitor, had no inhibitory effect on cell death induced by TNF in both RIPK1 knockdown and the negative control L929 cells (Figure 1A), indicating that both RIPK1 knockdown and the negative control cells dies through necroptotic pathway, but not apoptotic pathway. In addition, Nec-1, the allosteric inhibitor of RIPK1, fully blocked cell death in the negative control cells but had no inhibitory effect on RIPK1-knockdown cell death (Figure 1A), further confirming that cells dies through a RIPK1-independent manner.

Though RIPK3 could mediate RIPK1-dependent necroptosis, artificial activation of RIPK3 initiates necroptosis in a RIPK1-independent manner (Degterev et al., 2005; Moujalled et al., 2013; Kearney et al., 2014). Therefore, we next determined the role of RIPK3 in RIPK1-knockdown L929 cells necroptosis triggered by TNF. As shown in Figure 1B, the phosphorylation levels of RIPK3 and its substrate protein MLKL increased significantly in both RIPK1-knockdown and the negative control cells upon TNF stimulation, demonstrating that RIPK3-MLKL signaling pathway was activated by TNF, regardless of RIPK1 presence. Next, we determined the effect of genetic elimination of RIPK3 or MLKL on RIPK1-independent necroptosis, and the result shown in Figures 1C,D demonstrated that knockdown of RIPK3 or MLKL inhibited RIPK1-knockdown L929 cells necroptosis triggered by TNF, so RIPK3 is the target protein for RIPK1-independent necroptosis. Finally, we found that GSK’872, the inhibitor of RIPK3, blocked cell death in RIPK1-knockdown L929 cells (Figure 1E), further confirming the critical role of RIPK3-MLKL signaling pathway in RIPK1-independent necroptosis.

Collectively, our results demonstrate that RIPK3 and MLKL mediate RIPK1-independent necroptosis triggered by TNF.

### TRADD Is Essential for RIPK1-Independent Necroptosis in Response to TNF Stimulation

**FIGURE 2:**
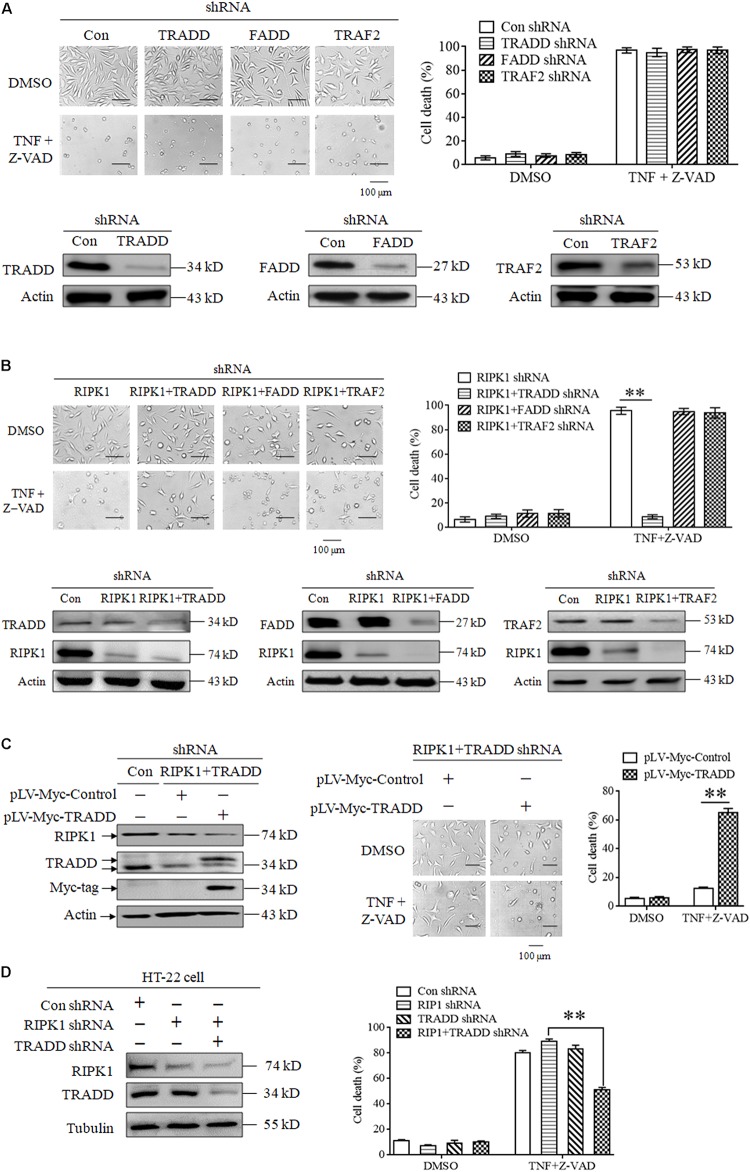
TRADD mediates RIPK1-independent necroptosis in response to TNF stimulation. **(A)** Knockdown of TRADD, FADD and TRAF2 has no inhibitory effect on TNF-induced L929 cells necroptosis in the presence of RIPK1. L929 cells were infected with TRADD shRNA, FADD shRNA, TRAF2 shRNA or the negative control shRNA len viruses for 48 h, and then treated with or without TNF plus Z-VAD for another 24 h. Western blotting was performed to evaluate the knockdown efficiency and actin was used as a loading control. Cell death was determined by microscopy (200× magnification) and quantified by measuring the proportion of propidium iodide-positive cells via flow cytometry. **(B)** TRADD knockdown inhibits TNF-induced necroptosis in RIPK1-knockdown L929 cells. Cells were infected with the indicated lentiviruses and then treated with or without TNF plus Z-VAD for 24 h. Western blotting was performed to evaluate the knockdown efficiency and actin was used as a loading control. Cell death was determined by microscopy (200× magnification) and quantified by measuring the proportion of propidium iodide-positive cells via flow cytometry. The cell death values (%) are shown as the mean ±SD ^∗∗^*P* < 0.01. **(C)** Restoration of TRADD expression recovers the sensitivity of L929 cells to necroptosis triggered by TNF plus Z-VAD. L929 cells were infected with the indicated lentiviruses and then treated with TNF plus Z-VAD for 24 h. The expression levels of RIPK1, TRADD, and Myc-tag were determined by western blotting, and actin was used as a loading control. Cell death was determined by microscopy (200× magnification) and quantified by measuring the proportion of propidium iodide-positive cells via flow cytometry. The cell death values (%) are shown as the mean ±SD ^∗∗^*P* < 0.01. **(D)** TRADD knockdown inhibits TNF-induced necroptosis in RIPK1-knockdown HT-22 cells. Cells were infected with the indicated lentiviruses and then treated with or without TNF plus Z-VAD for 24 h. The knockdown efficiency was verified by western blotting, and tubulin was used as a loading control. Cell death was determined by measuring the proportion of propidium iodide-positive cells via flow cytometry. The cell death values (%) are shown as the mean ±SD ^∗∗^*P* < 0.01.

Though RIPK3 mediates RIPK1-independent necroptosis, it is unclear how to transduce the necroptotic signal from TNFR1 to RIPK3 because RIPK3 has no death domain to directly bind TNFR1. In this study, we explored the role of some adaptor proteins, including TRADD, FADD and TRAF2, in RIPK1-independent necroptosis. Firstly, we explored the effect of TRADD, FADD, and TRAF2 knockdown on TNF-induced necroptosis. As shown in Figure 2A, knockdown of TRADD, FADD or TRAF2 had no inhibitory effect on L929 cells necroptosis induced by TNF plus Z-VAD, indicating that TRADD, FADD and TRAF2 are dispensable for RIPK1-dependent necroptosis induction. Next, we evaluated the effect of TRADD, FADD, and TRAF2 on RIPK1-independent necroptosis in response to TNF stimulation. As shown in Figure 2B, TNF induced a substantial amount of RIPK1-knockdown L929 cell death, whereas the RIPK1-independent cell death was inhibited by simultaneous knockdown of TRADD, but not FADD or TRAF2, indicating that TRADD is essential for RIPK1-independent necroptosis. In addition, restoration of TRADD protein expression in RIPK1 and TRADD double-knockdown L929 cells led to the recovery of cell sensitivity to TNF-induced necroptosis (Figure 2C), further supporting the critical role of TRADD in RIPK1-independent necroptosis. Finally, we verified the role of TRADD in RIPK1-independent necroptosis in HT-22 cells, another kind of cellular model for necroptosis research. As shown in Figure 2D, RIPK1 knockdown did not inhibit HT-22 cell death induced by TNF plus Z-VAD, whereas the simultaneous knockdown of TRADD partially protected RIPK1-knockdown HT-22 cells from TNF-induced cell death. Moreover, TRADD knockdown alone did not inhibit HT-22 cell death induced by TNF plus Z-VAD in the presence of RIPK1, so TRADD plays a critical role in mediating RIPK1-knockdown HT-22 cell necroptosis in response to TNF stimulation.

In conclusion, our results demonstrate that TRADD functions as a critical target protein for RIPK1-independent necroptosis induction.

### TRADD Is Essential for TNF-Induced Activation of RIPK3-MLKL Signaling Pathway in RIPK1-Knockdown L929 Cells

**FIGURE 3:**
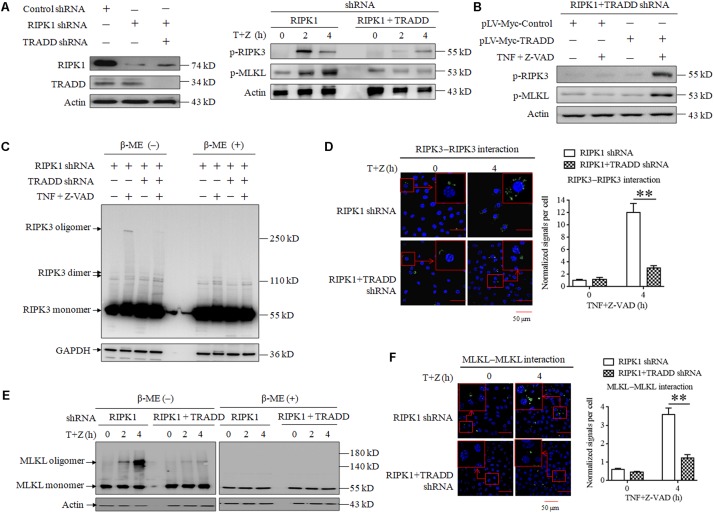
TRADD is essential for RIPK3–MLKL signaling pathway activation triggered by TNF. **(A)** TRADD knockdown suppresses phosphorylation of RIPK3 and MLKL induced by TNF plus Z-VAD in the absence of RIPK1. L929 cells were infected with RIPK1 shRNA lentivirus with or without TRADD shRNA lentivirus for 48 h, and then treated with or without TNF plus Z-VAD (T+Z) for the indicated length of time. Western blot was used to detect the phosphorylation level of RIPK3 and MLKL (p-RIPK1 and p-MLKL) and the protein level of RIPK1 and TRADD. Actin was used as a loading control. **(B)** Restoration of TRADD expression recovers the activation of RIPK3-MLKL signaling pathway in the absence of RIPK1. RIPK1 and TRADD double-knockdown L929 cells were infected with pLV-Myc-TRADD and the negative control lentiviruses for 48 h, and then treated with or without TNF plus Z-VAD for 4 h. Western blotting was performed to evaluate the phosphorylation levels of RIPK3 and MLKL (p-RIPK3 and p-MLKL), and actin was used as a loading control. In addition, the expression level of TRADD, RIPK1, and Myc-tag was verified in Figure 2C. **(C,E)** TRADD knockdown suppresses TNF-induced RIPK3 and MLKL oligomerization in the absence of RIPK1. RIPK1-knockdown or RIPK1 and TRADD-double knockdown L929 cells were treated with or without TNF plus Z-VAD (T+Z), and lysed with cell lysis buffer containing or not containing β-ME. Western blotting was used to detect the monomer and oligomer of RIPK3 (Figure 3C) and MLKL (Figure 3E). Actin was used as a loading control. **(D,F)** TRADD is essential for the homo-interaction of RIPK3 or MLKL in response to TNF stimulation. RIPK1 knockdown or RIPK1 and TRADD double-knockdown L929 cells were treated with TNF plus Z-VAD (T+Z) for the indicated length of time, and the homodimers of RIPK3 or MLKL were detected with a Duolink proximity ligation assay and confocal microscopy. All Duolink proximity ligation experiments were performed at least three times, and representative results are shown. The normalized signal per cell is shown as the mean ±SD of three separate experiments (*n* = 3). ^∗∗^*P* < 0.01.

As TRADD is essential for RIPK1-independent necroptosis initiating by RIPK3, we next explored the role of TRADD in RIPK3-MLKL signaling transduction. As shown in Figure 3A, the phosphorylation levels of RIPK3 and MLKL significantly increased in RIPK1-knockdown L929 cells but not RIPK1 and TRADD double-knockdown L929 cells in response to TNF stimulation. Moreover, restoration of TRADD protein expression recovered the phosphorylation of RIPK3 and MLKL in RIPK1 and TRADD double-knockdown L929 cells after TNF treatment (Figure 3B). Therefore, our results demonstrate that TRADD is essential for RIPK3-MLKL signaling pathway activation in the process of RIPK1-independent necroptosis. It has been reported that RIPK3 phosphorylation derived from the intramolecular reaction between RIPK3 dimers or oligomers (Orozco et al., 2014; Wu et al., 2014), so we detected the effect of TRADD on RIPK3 oligomerization in RIPK1-knockdown L929 cells. As shown in Figure 3C, three specific bands were detected on PVDF membrane by using RIPK3 antibody, which represented RIPK3 monomers (55 kD), dimer (approximate 110 kD) and oligomer (above 250 kD), respectively. There was a substantial amount of RIPK3 oligomers in RIPK1-knockdown cells but their levels were much lower in RIPK1 and TRADD double-knockdown cells in response to TNF stimulation. Therefore, our results demonstrate that TRADD is essential for RIPK3 oligomerization. As the RIPK3 oligomer could be detected in non-reduced protein samples (without β-Mercaptoethanol, β-ME) but not in the reduced protein samples (containing β-ME), the RIPK3 oligomer in our data is formed by the crosslinking of the oxidized disulfide bond and is not a non-specific band. Unlike RIPK3 oligomer, RIPK3 dimer was detected in the protein samples containing β-ME, suggesting that the formation of RIPK3 dimer does not depend on the crosslinking of the disulfide bond. Moreover, TRADD knockdown also slightly suppressed the formation of RIPK3 dimer, suggesting that TRADD is also essential for RIPK3 dimerization in the absence of RIPK1. In addition, we determined RIPK3 homo-interaction by performing a Duolink proximity ligation assay (PLA), which can detect the hetero-dimer between different proteins and home-dimer between the same proteins. As shown in Figure 3D, the green sports, which represent the homo-interaction of RIPK3 proteins, increased in RIPK1-knockdown L929 cells but not the RIPK1 and TRADD double-knockdown L929 cells after TNF plus Z-VAD treatment. Moreover, we also analyzed the PLA signals representing the protein interaction, and found that the normalized PLA signals increased in the RIPK1-knockdown L929 cells but not the RIPK1 and TRADD double-knockdown L929 cells treated with TNF plus Z-VAD (Figure 3D), further confirming that TRADD is critical for RIPK3 homo-interaction in response to TNF stimulation.

Because phosphorylated MLKL oligomerizes via disulfide bonds (Chen et al., 2014; Galluzzi et al., 2014; Wang et al., 2014), we next detected the effect of TRADD on MLKL oligomer formation. As shown in Figure 3E, MLKL oligomers increased in RIPK1-knockdown L929 cells with a time-dependent manner in response to TNF plus Z-VAD treatment, but TRADD knockdown almost fully suppressed the formation of MLKL oligomers. Consistent with the previous study (Chen et al., 2014; Galluzzi et al., 2014), MLKL oligomers were detected in protein samples without β-ME. In addition, we also determined MLKL homo-interaction by using a Duolink PLA assay, and the results demonstrated that the Duolink PLA signal for MLKL homodimerization in the RIPK1-knockdown cells was significantly higher than that in RIPK1 and TRADD double-knockdown cells after TNF plus Z-VAD treatment (Figure 3F), further confirming the critical role of TRADD in MLKL oligomerization.

In conclusion, TRADD is critical for RIPK3 and MLKL phosphorylation and oligomerization in RIPK1-knockdown L929 cells in response to TNF stimulation.

### RIPK1 Knockdown Facilitates the Interaction Between TRADD and RIPK3 in Response to TNF Stimulation

**FIGURE 4:**
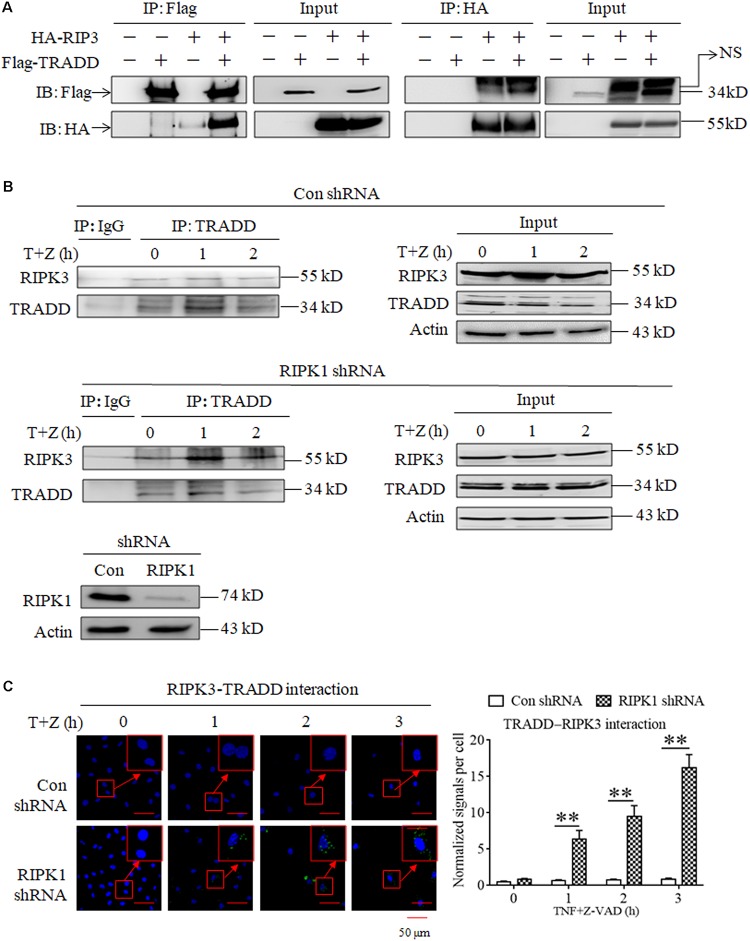
RIPK1 knockdown facilitates the interaction between RIPK3 and TRADD in response to TNF stimulation. **(A)** The interaction between ectopically expressed TRADD and RIPK3. 293T cells were transfected with the indicated mammalian expression vectors. After 48 h, cells were collected and lyzed, and cell lysates were then subjected to immunoprecipitation (IP) with anti-FLAG or anti-HA antibody, followed by SDS-PAGE and immunoblot (IB) analysis with the indicated antibody. **(B)** The interaction between RIPK3 and TRADD significantly increased in RIPK1-knockdown L929 cells treated with TNF plus Z-VAD. RIPK1-knockdown and the negative control L929 cells were treated with TNF plus Z-VAD (T+Z) for the indicated length of time, and the interaction between RIPK3 and TRADD was determined by immunoprecipitation and western blot. Actin was used as a loading control. **(C)** TNF induces the interaction between RIPK3 and TRADD in RIPK1-knockdown L929 cells. RIPK1-knockdown and the negative control L929 cells were treated with TNF plus Z-VAD (T+Z) for the indicated length of time, and RIPK3-TRADD heterodimer were detected with a Duolink proximity ligation assay and confocal microscopy. All Duolink proximity ligation experiments were performed at least three times, and representative results are shown. The normalized signal per cell is shown as the mean ±SD of three separate experiments (*n* = 3). ^∗∗^*P* < 0.01. The RIPK1 knockdown efficiency was verified in Figure 1A.

As TRADD is essential for RIPK3 activation in RIPK1-independent necroptosis, we next determined whether TRADD could directly bind RIPK3. As shown in Figure 4A, we detected the interaction between the Flag-TRADD and HA-RIPK3 proteins ectopically expressed in 293T cells, and found that immunoprecipitation with Flag-tag antibody could pull down HA-RIPK3, and vice versa, indicating that TRADD could bind RIPK3 to form protein complex. Moreover, we determined the interaction between TRADD and RIPK3 in the process of TNF-induced necroptosis and found that the interaction between TRADD and RIPK3 increased in RIPK1-knockdown L929 cells but not the negative control L929 cells upon TNF stimulation (Figure 4B), suggesting that RIPK1 knockdown facilitates the interaction between TRADD and RIPK3. In addition, we determined the heterodimer of TRADD and RIPK3 by performing a Duolink proximity ligation assay, and the results shown in Figure 4C demonstrated that the PLA signals for TRADD and RIPK3 interaction increased in a time-dependent manner in RIPK1-knockdown, but not the negative control, L929 cells after TNF plus Z-VAD treatment, further confirming that RIPK1 knockdown facilitates the interaction between TRADD and RIPK3 in response to TNF stimulation.

In conclusion, our results demonstrate that RIPK1 knockdown facilitates the formation of TRADD and RIPK3 heterodimer upon TNF stimulation.

### ROS Accumulate in a TRADD-Dependent Manner and Contribute to TNF-Induced Necroptosis in RIPK1-Knockdown L929 Cells

**FIGURE 5:**
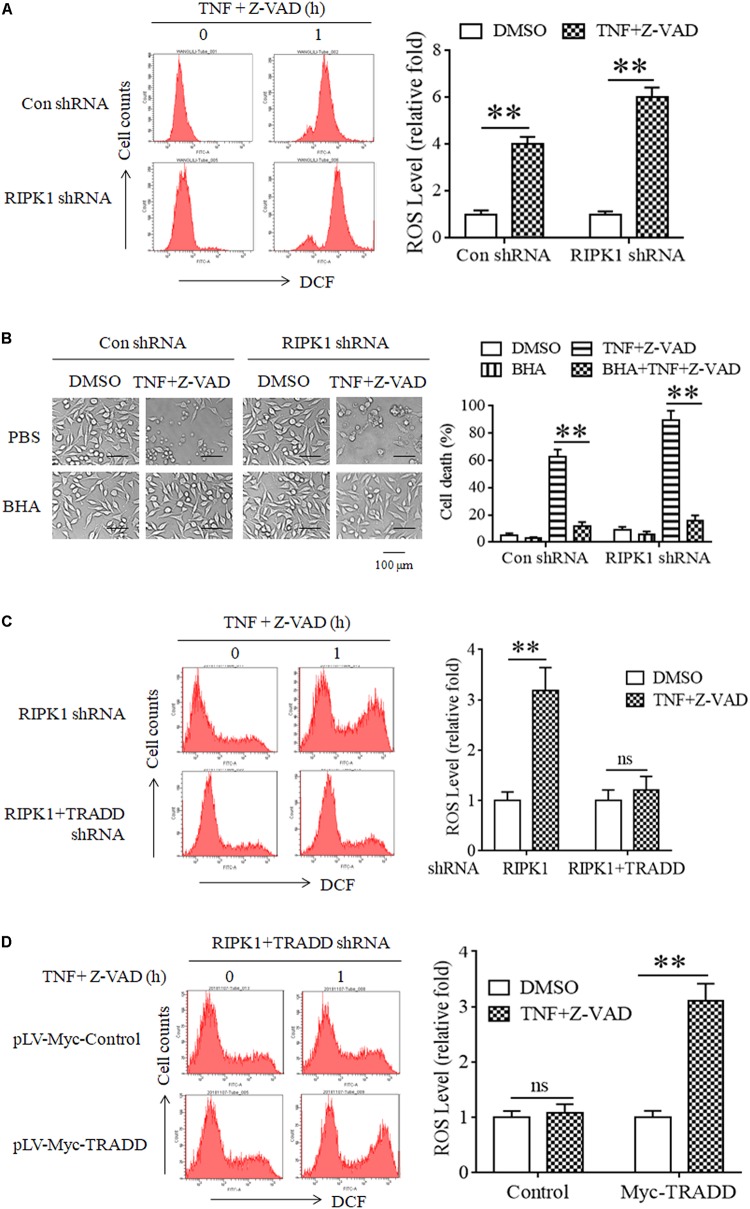
ROS accumulate in a TRADD-dependent manner and mediate necroptosis in RIPK1-knockdown L929 cells upon TNF stimulation. **(A)** ROS accumulate in RIPK1-knockdown and the negative control L929 cells after TNF plus Z-VAD treatment. RIPK1-knockdown and the negative control L929 cells were treated with TNF plus Z-VAD for 1 h, and ROS level was measured by flow cytometry and normalized by comparing the fluorescence representing ROS level in different groups with that in the control group (treated with TNF plus Z-VAD for 0 h). The RIPK1 knockdown efficiency was verified in Figure 1A. ^∗∗^*P* < 0.01. **(B)** Clearance of ROS with Butyl hydroxyl anisd (BHA) inhibits RIPK1-dependent and independent necroptosis. RIPK1-knockdown and the negative control cells were treated with TNF plus Z-VAD in the presence and absence of BHA for 24 h, and cell death was determined by microscopy (200× magnification) and quantified by measuring the proportion of propidium iodide-positive cells via flow cytometry. The RIPK1 knockdown efficiency was verified in Figure 1A. ^∗∗^*P* < 0.01. **(C)** TRADD knockdown suppresses ROS accumulation in the absence of RIPK1. RIPK1-knockdown or RIPK1 and TRADD double-knockdown cells were treated with TNF plus Z-VAD for 1 h, and ROS level was measured by flow cytometry and normalized by comparing the fluorescence representing ROS level in different groups with that in the control group (treated with TNF plus Z-VAD for 0 h). The knockdown efficiency of RIPK1 and TRADD was verified in Figure 3A. ^∗∗^*P* < 0.01. ns, no significance. **(D)** Restoration of TRADD expression recovers the sensitivity of L929 cells to TNF-induced ROS accumulation. RIPK1 and TRADD double-knockdown L929 cells were infected with pLV-Myc-TRADD and the negative control lentiviruses for 48 h, and then treated with or without TNF plus Z-VAD for 1 h. Cellular ROS level was measured by flow cytometry and normalized by comparing the fluorescence representing ROS level in different groups with that in the control group (treated with TNF plus Z-VAD for 0 h). The protein level of RIPK1 and TRADD was verified in Figure 2C. ^∗∗^*P* < 0.01. ns, no significance. All the ROS levels (relative fold) are shown as the mean ±SD of three separate experiments (*n* = 3) and they were analyzed by a two-tailed *t*-test.

It has been reported that RIPK3 phosphorylation facilitates cellular ROS accumulation, which is critical for TNF-induced L929 cell necroptosis (Schenk and Fulda, 2015; Zhang et al., 2017). As TRADD is essential for RIPK3 phosphorylation in RIPK1-independent necroptosis, we next determined the effect of TRADD on ROS accumulation. As shown in Figure 5A, ROS levels significantly increased in both RIPK1-knockdown and the negative control L929 cells in response to TNF stimulation. Moreover, Butyl hydroxyl anisd (BHA), the antioxidant reagent, significantly inhibited both RIPK1-dependent and independent necroptosis (Figure 5B). Therefore, our data demonstrate that ROS also accumulate in RIPK1-knockdown L929 cells and mediated RIPK1-independent necroptosis. However, TNF-induced ROS accumulation in RIPK1-knockdown L929 cells was inhibited by the simultaneous knockdown of TRADD (Figure 5C). Moreover, restoration of TRADD expression in TRADD and RIPK1 double-knockdown L929 cells recovered ROS accumulation induce by TNF plus Z-VAD (Figure 5D). Therefore, these data demonstrate that TRADD is essential for TNF-induced ROS accumulation in the absence of RIPK1.

In conclusion, ROS accumulate in a TRADD-dependent manner and contribute to RIPK1-independent necroptosis in response to TNF stimulation.

## Discussion

In this study, TRADD was identified as a target protein for TNF-induced necroptosis in RIPK1-knockdown L929 and HT-22 cells. As the firstly identified adaptor protein that directly binds TNFR via death domain, TRADD recruits several other proteins, including RIPK1 and TRAF2, to form protein complex and then mediates the signal transduction downstream of TNFR ligation (Hsu et al., 1995, 1996; Ermolaeva et al., 2008; Pobezinskaya and Liu, 2012). It has been reported that TRADD is required for TNF-induced apoptosis, but RIPK1 is critical for necroptosis (Micheau and Tschopp, 2003; Zheng et al., 2006; Vanlangenakker et al., 2011). Therefore, genetic elimination of TRADD has no inhibitory effect on TNF-induced necroptosis in L929 and Jurkat cells (Zheng et al., 2006; Vanlangenakker et al., 2011), which was also confirmed in our study. However, we found that TRADD knockdown blocked necroptosis of RIPK1-knockdown L929 and HT-22 cells in response to TNF stimulation. Moreover, TRADD bound RIPK3 to form protein complex, which facilitated RIPK3 oligomerization and the subsequent phosphorylation. Therefore, TRADD acts as the partner of RIPK3 to initiate necroptosis in the absence of RIPK1. It has been reported that RIPK1 knockdown facilitates necroptosis as well as apoptosis in mouse embryo development, so RIPK1 deletion induces perinatal lethality. Though the simultaneous deletion of TRADD provide no survival advantage to RIPK1^–/–^ animals, it is sufficient to reduce the systemic cell death and inflammation (Anderton et al., 2019; Dowling et al., 2019). Therefore, TRADD may also function to mediate RIPK1-independent necroptosis *in vivo* (Anderton et al., 2019; Dowling et al., 2019). Previous study indicates that TRADD competes with RIPK1 to bind TNFR (Zheng et al., 2006), so it is possible to raise the hypothesis that TRADD and RIPK1 compete to bind RIPK3 to form functional protein complex in necroptosis induction. As RIPK1 binds RIPK3 through their shared RHIM domain, which is more optimal than other protein domain in mediating protein interaction, it is more capable for RIPK1 than TRADD to be recruited by RIPK3 to initiate necroptosis (Wu et al., 2014; Baker et al., 2018). Therefore, TRADD is dispensable for necroptosis induction in the presence of RIPK1, and knockdown of RIPK1 facilitates the interaction between TRADD and RIPK3, resulting in TRADD-dependent necroptosis. However, TRADD has been identified to be essential for necroptotic MEF cell death induced by TNF plus cycloheximide and Z-VAD in the presence of RIPK1 (Ermolaeva et al., 2008; Pobezinskaya et al., 2008). Moreover, TRADD knockdown also inhibits necroptosis of MEF cells with ectopical expression of RIPK3 after TNF plus smac mimetic treatment (Moujalled et al., 2013). These reports demonstrate that TRADD is also critical for TNF-induced necroptosis in the presence of RIPK1, which is not compatible with our data and some of the previous reports. It has been reported that TRADD mediates the interaction between TNFR with other adaptor proteins containing death domain, including RIPK1, in MEF cells, so depletion of TRADD almost fully blocks the signaling transduction downstream of TNFR1 ligation, including apoptosis, necroptosis and NFκB pathway activation (Ermolaeva et al., 2008; Pobezinskaya et al., 2008). However, depletion of TRADD only partially suppresses the interaction between TNFR and RIPK1, as well as the subsequent apoptosis and NFκB activation in peritoneal macrophages in response to TNF stimulation (Ermolaeva et al., 2008; Pobezinskaya et al., 2008). Comparing with MEF cells, macrophages have a higher expression of RIPK1, which may compensate for the effect of TRADD to form a signaling complex to mediate signaling transduction downstream of TNFR1 ligation (Ermolaeva et al., 2008; Pobezinskaya et al., 2008; Pobezinskaya and Liu, 2012). Therefore, TRADD seems to be more critical in cells with low expression of RIPK1. In addition, RIPK1 was also highly expressed in L929 cells (Vercammen et al., 1998; Humphries et al., 2015), which guarantees the interaction between RIPK1 and TNFR in the absence of TRADD, so TRADD knockdown has no inhibitory effect on normal L929 cell necroptosis induced by TNFR ligation.

In this study, we found that RIPK3 formed oligomer through crosslinking of oxidized disulfide bound because RIPK3 oligomer could only be detected in non-reduced protein samples. Moreover, ROS accumulated in response to TNF stimulation and mediated necroptosis in RIPK1-knockdown L929 cells. Therefore, it is reasonable to presume that RIPK3 oligomer derived from ROS-oxidized disulfide bond. Similar to our result, RIPK1 oligomer only can be detected in non-reduced protein samples (Zhang et al., 2017). Mechanistic study discovers that three cysteins in RIPK1 protein can be oxidized by ROS and form intermolecular disulfide bonds, which is required for forming RIPK1 oligomers, so RIPK1 forms oligomers via oxidized disulfide bonds, but not RHIM domain (Zhang et al., 2017). As RIPK1 oligomer promotes RIPK1 autophosphorylation through intramolecular reaction, RIPK3 oligomer in our study may facilitate RIPK3 autophosphorylation through the same mechanism, which may explain for the mechanism of RIPK3 activation in the absence of RIPK1. However, the chemically enforced dimerization of RIPK3 enable RIPK3 in the dimer to recruit free RIPK3 into the protein complex and form RIPK3 oligomer via RHIM domain (Orozco et al., 2014; Wu et al., 2014). Therefore, RIPK3 may form oligomer through two different mechanisms. In addition, the artificial oligomer of RIPK3 forms on the basis of chemically enforced dimerization of RIPK3 ectopically expressed in 293T cells, which does not require TNF stimulation and ROS accumulation (Orozco et al., 2014; Wu et al., 2014). However, RIPK3 oligomer in our study formed on the basis of intermolecular interaction mediated by crosslinking of oxidized disulfide bonds, which required TNF stimulation and ROS accumulation. Therefore, the different cellular model and stimuli for necroptosis induction used in our study and the previous sports may account for the discrepancy between these two distinct RIPK3 oligomers.

In summary, TRADD was identified as a target protein for TNF-induced necroptosis in the absence of RIPK1. The mechanistic study demonstrated that TRADD bounds RIPK3 to form new protein complex, and promoted RIPK3 activation via facilitating RIPK3 oligomerization. Therefore, our study discovered the exact role of TRADD, RIPK1, and RIPK3 in necroptosis induction and illuminated their coordinative and competitive relationship in initiating necroptotic signal transduction in response to TNF stimulation.

## Materials and Methods

### Cells and Reagents

L929 fibrosarcoma cells, HT-22 mouse neuronal cells and 293T cells were purchased from the Cell Culture Center, Beijing Institute of Basic Medical Science of the Chinese Academy of Medical Sciences (Beijing, China). 293TN cells were obtained from System Biosciences (SBI, Mountain View, CA, United States). The cells were cultured in Dulbecco’s modified Eagle medium (Gibco, Grand Island, NY, United States) containing 10% fetal bovine serum (FBS; Kangyuan Biology, China). Necrostatin-1 (Nec-1, 50 μM) and Z-VAD-FMK (20 μM) were purchased from Medchem Express (Beijing, China). GSK’872 was obtained from Biovision (Milpitas, CA, United States). Tumor necrosis factor (100 ng/mL) was obtained from GeneScript (Nanjing, China). Butyl hydroxyl anisd (BHA, 100 μM), β-mercaptoethanol (β-ME) and propidium iodide (PI) were purchased from Sigma-Aldrich (St. Louis, MO, United States).

### Cell Death Analysis

Cell death analysis was performed according to the method described by Chang et al. (2017). Briefly cell death was determined by microscopy (at a magnification of 200×), and cell death ratio was measured by flow cytometry.

### Western Blotting

Western blotting experiment was described previously (Zhao et al., 2019), and the detection of RIPK3 or MLKL oligomers was performed according to the method described by Wang et al. (2019). The following antibodies were used for the experiments: anti-FADD (1:1000, 610399) and anti-RIPK1 (1:3000, 610458) (BD Transduction Laboratories, San Jose, CA); anti-phospho-RIPK3 (1:1000, ab195117), anti-MLKL (1:1000, ab172868), anti-phospho-MLKL (1:1000, ab196436) (Abcam, Cambridge, MA, United States); anti-RIPK1 (1:3000, 3493), anti-RIPK3 (1:3000, 15828), anti-Myc tag (1:2000, 2276), anti-HA tag (1:2000, 3724), anti-Flag tag (1:2000, 14793), anti-TRAF2 (1:1000, 4724), anti-phospho-RIPK1 (1:1500, 31122) and anti-phospho-RIPK3 (1:1000, 57220) [Cell Signaling Technology (CST), Beverly, MA]; anti-TRADD (1:1000, ABP52634) and anti-GAPDH (1:1500, ABP52783) (Abbkine, Redlands, CA, United States); anti-β-actin (1:3000, A5441) (Sigma-Aldrich). All western blot assays were performed three times, and the representative results are shown.

### Immunoprecipitation

Immunoprecipitation was described previously (Wang et al., 2019). Flag (14793, CST) and HA tag (3724, CST) antibody were used to pull down the Flag-TRADD or HA-RIP3 ectopically expressed in 293T cells, and TRADD antibody (sc-46653, Santa Cruz Biotechnology) was used to pull down TRADD protein endogenously expressed in L929 cells.

### Gene Repression

Gene repression was described previously (Wang et al., 2019), and DNA sequences targeting the following specific genes were inserted into the lenti-shRNA vectors: mouse RIPK1 (5′-GCATTGTCCTTTGGGCAAT-3′), mouse RIPK3 (5′-GCTGAGTTGGTAGACAAGA-3′), mouse TRADD (5′-GCAAAGACCCTCTAAGTACCCGGAC-3′ and 5′-CCAGCA GTTCAGTGTTTGAAA-3′), mouse FADD (5′-CCATCT CAGTTAAGATCACTTGGTT-3′), mouse TRAF2 (5′-GTGA TTAAATGTTGAGATGTCTGTG-3′), mouse MLKL (5′-AGA TCCAGTTCAACGATATAT-3′) and negative control (5′-TTC TCCGAACGTGTCACGT-3′).

### Ectopic Expression of TRADD and RIPK3

Mouse TRADD cDNA was cloned into the pEasy-Blunt M3 vector (TransGen Biology, Beijing) to express Flag-tag TRADD, and verified by DNA sequencing. HA-tagged RIPK3 plasmid (MG51069-NY) was purchased from Sino biological (Beijing, China). The plasmids were transfected into 293T cells using Chemifect transfection reagent (Fengrui Biotechnology) to overexpress the target genes with different tag sequences. The protein levels of Flag and HA tags were measured by western blot analysis. The pLV-Myc-TRADD plasmid was obtained from Cyagen Biosciences (Guangzhou, China), and co-transfected into 293TN cells (SBI) with second-generation packaging systems to produce lentivirus, which mediates TRADD ectopical expression after infecting L929 cells.

### Duolink Proximity Ligation Assay

Duolink proximity ligation assay was described previously (Wang et al., 2019). The antibodies were all used at 1:80, including rabbit anti-TRADD (abp52634, Abbkine) and mouse anti-RIPK3 (sc-374639, Santa Cruz) (for detecting TRADD-RIPK3 heterodimer) antibodies, mouse anti-RIPK3 (sc-374639, Santa Cruz) and rabbit anti-RIPK3 (15828, CST) (for detecting RIPK3 homodimer) antibodies, or mouse anti-MLKL (66675-1-Ig, Proteintech, Rosemont, IL) and rabbit anti-MLKL (ab172868, abcam) (for detecting MLKL homodimer) antibodies. Three independent experiments were performed, and the representative results are shown.

### Measurement of Reactive Oxygen Species

Cellular ROS were measured with a Reactive Oxygen Species Assay Kit (Beyotime Institute of Biotechnology). Briefly, cells were collected by trypsinization and washed with PBS, then incubated with 5 μM DCFH-DA for 30 min. After washing with PBS, the cells were analyzed by flow cytometry (FACS Calibur) at an excitation wavelength of 480 nm and an emission wavelength of 525 nm. Twenty thousand stained cells were analyzed with flow cytometry for each measurement. The ROS fold changes were normalized by comparing the mean geometry fluorescence in all groups with that in the group treated with DMSO (the negative control of TNF plus Z-VAD stimulation).

### Statistical Analysis

GraphPad prism 7 software was used to analyze the data and construct statistical graphs. Statistical significance was analyzed by performing a two-tailed Student’s *t*-test or one-way ANOVA test and defined as *P* < 0.01. All experiments were repeated at least three times, and the data are expressed as the mean ±SD from representative experiments.

## Data Availability Statement

The datasets generated for this study can be found in the NCBI Reference Sequences: NM_001033161.2, NM_009068.3, and NM_019955.2.

## Author Contributions

LW, XC, and JF prepared all the figures. JY collected some data. GC designed all the experiments and wrote the manuscript. All authors reviewed the manuscript.

## Conflict of Interest

JY was employed by company Beijing Zhendandingtai Biotechnology Co., Ltd.

The remaining authors declare that the research was conducted in the absence of any commercial or financial relationships that could be construed as a potential conflict of interest.

## Abbreviations

FADD: fas-associated death domain
MLKL: mixed-lineage kinase domain-like protein
RIPK1: receptor-interacting serine/threonine-protein kinase 1
RIPK3: receptor-interacting serine/threonine-protein kinase 3
ROS: reactive oxygen species
TNFR1: tumor necrosis factor receptor 1
TRADD: TNF receptor type 1-associated death domain protein
TRAF2: TNFR-associated factor 2.
